# Microbial community structures and important taxa across oxygen gradients in the Andaman Sea and eastern Bay of Bengal epipelagic waters

**DOI:** 10.3389/fmicb.2022.1041521

**Published:** 2022-11-02

**Authors:** Ruoyu Guo, Xiao Ma, Jingjing Zhang, Chenggang Liu, Chit Aung Thu, Tun Naing Win, Nyan Lin Aung, Hlaing Swe Win, Sanda Naing, Hongliang Li, Feng Zhou, Pengbin Wang

**Affiliations:** ^1^Key Laboratory of Marine Ecosystem Dynamics, Second Institute of Oceanography, Ministry of Natural Resources, Hangzhou, China; ^2^Observation and Research Station of Yangtze River Delta Marine Ecosystems, Ministry of Natural Resources, Zhoushan, China; ^3^State Key Laboratory of Satellite Ocean Environment Dynamics, Second Institute of Oceanography, Ministry of Natural Resources, Hangzhou, China; ^4^Research and Development Section, Department of Fisheries, Naypyidaw, Myanmar; ^5^Department of Meteorology and Hydrology, Ministry of Transport and Communication, Naypyidaw, Myanmar; ^6^Environmental Conservation Department, Ministry of Natural Resources and Environmental Conservation, Naypyidaw, Myanmar; ^7^National Analytical Laboratory, Department of Research in Innovation, Ministry of Education, Naypyidaw, Myanmar; ^8^Port and Harbour Engineering Department, Myanmar Maritime University, Thanlyin, Myanmar

**Keywords:** oxygen minimum zone, keystone taxa, bioindicator taxa, Andaman Sea, Bay of Bengal

## Abstract

In oceanic oxygen minimum zones (OMZs), the abundances of aerobic organisms significantly decrease and energy shifts from higher trophic levels to microorganisms, while the microbial communities become critical drivers of marine biogeochemical cycling activities. However, little is known of the microbial ecology of the Andaman Sea and eastern Bay of Bengal (BoB) OMZs. In the present study, a total of 131 samples which from the Andaman Sea and eastern BoB epipelagic waters were analyzed. The microbial community distribution patterns across oxygen gradients, including oxygenic zones (OZs, dissolved oxygen [DO] ≥ 2 mg/L), oxygen limited zones (OLZs, 0.7 mg/L < DO < 2 mg/L), and OMZs (DO ≤ 0.7 mg/L), were investigated. Mantel tests and Spearman’s correlation analysis revealed that DO was the most important driver of microbial community structures among several environmental factors. Microbial diversity, richness, and evenness were highest in the OLZs and lowest in the OZs. The microbial community compositions of OZ and OMZ waters were significantly different. Random forest analysis revealed 24 bioindicator taxa that differentiated OZ, OLZ, and OMZ water communities. These bioindicator taxa included Burkholderiaceae, HOC36, SAR11 Clade IV, Thioglobaceae, Nitrospinaceae, SAR86, and UBA10353. Further, co-occurrence network analysis revealed that SAR202, AEGEAN-169, UBA10353, SAR406, and Rhodobacteraceae were keystone taxa among the entire interaction network of the microbial communities. Functional prediction further indicated that the relative abundances of microbial populations involved in nitrogen and sulfur cycling were higher in OMZs. Several microbial taxa, including the Thioglobaceae, Nitrospinaceae, SAR202, SAR406, WPS-2, UBA10353, and Woeseiaceae, may be involved in nitrogen and/or sulfur cycling, while also contributing to oxygen consumption in these waters. This study consequently provides new insights into the microbial community structures and potentially important taxa that contribute to oxygen consumption in the Andaman Sea and eastern BoB OMZ.

## Introduction

Dissolved oxygen (DO) concentration is one of most important factors that shapes community structures and functions in marine ecosystems (Vaquer-Sunyer and Duarte, 2008; Beman and Carolan, 2013). As deoxygenation intensifies in marine ecosystems, aerobic organism abundances significantly decrease and biogeochemical processes are altered, leading to energetic shifts from higher trophic levels to microorganisms (Diaz and Rosenberg, 2008; Wright et al., 2012). Ocean regions where oxygen decreases to very low concentrations and then rises again with increasing depth are termed oxygen minimum zones (OMZs) (Paulmier and Ruiz-Pino, 2009). Previous studies have revealed numerous microbial taxa and microbial-mediated biogeochemical cycling patterns associated with global OMZs (Walsh et al., 2009; Wright et al., 2012; Padilla et al., 2016; Long et al., 2021). Indeed, OMZs harbor unique microbial taxa and community compositions (Beman and Carolan, 2013; Bush et al., 2017). Hence, microbial communities that mediate biogeochemical cycling in OMZs are distinct from those in oxygenic regions (Beman and Carolan, 2013; Bertagnolli and Stewart, 2018). Nitrogen and/or sulfur cycling activities generally increase in OMZs (Ulloa et al., 2012; Wright et al., 2012; Penn et al., 2019). Moreover, microbial communities mediate numerous biogeochemical feedbacks in OMZs that can exacerbate or ameliorate deoxygenation by participating in nitrogen, sulfur, and carbon cycles (Levin, 2018). In recent years, microbial studies have significantly improved our understanding of the ecology and biogeochemical cycling within OMZs.

Oxygen minimum zones typically occur at water depths of 100–1500 m underlying surface waters. Four permanent OMZs have been identified globally including the eastern tropical North Pacific (ETNP), the eastern tropical South Pacific (ETSP), the Arabian Sea, and the Bay of Bengal (BoB) (Diaz and Rosenberg, 2008; Paulmier and Ruiz-Pino, 2009; Lam and Kuypers, 2011; Breitburg et al., 2018). However, coastal eutrophication and climate change have led to OMZs expanding and shoaling in past decades, and these processes remain ongoing (Diaz and Rosenberg, 2008; Deutsch et al., 2011; Schmidtko et al., 2017; Breitburg et al., 2018; Oschlies, 2021). The expansion and shoaling of OMZs can potentially alter the microbial communities and microbially-mediated biogeochemical cycling activities in these regions (Gilly et al., 2013; Bertagnolli and Stewart, 2018).

The Andaman Sea, located in the northeastern Indian Ocean, is a semi-closed marginal sea, and is bounded by Myanmar to the north, in addition to Thailand and Malaysia in the east, while being partly isolated by the Andaman and Nicobar Islands from the BoB (Dutta et al., 2007). The Andaman Sea is connected to the eastern BoB through shallow passages including the Preparis Channel in the north, the Ten Degree Channel, and the Great Channel in the south (Jithin and Francis, 2020). A large freshwater influx and seasonal monsoon winds lead to the region containing the Andaman Sea and BoB being distinct from other water bodies in tropical regions (Han and Mccreary, 2001; Mahadevan, 2016). However, investigations of the microbial ecology in the Andaman Sea and BoB in addition to its associations with oxygen concentration effects remain limited. A few microbial diversity studies have been conducted focusing on the BoB OMZ (Rajpathak et al., 2018; Fernandes et al., 2020; Vijayan et al., 2020; Gu et al., 2022). Specifically, 16S rRNA gene sequencing investigations, analysis of specific functional genes, culture-based methods, and metagenomic analyses have been used to evaluate BoB OMZ microbial communities (Bristow et al., 2016; Rajpathak et al., 2018; Fernandes et al., 2020; Vijayan et al., 2020; Gu et al., 2022). Microbial taxa that potentially function in nitrogen and/or sulfur cycling in the BoB OMZs include the SAR11, Pelagibacteraceae, and Caulobacteraceae groups (Rajpathak et al., 2018; Fernandes et al., 2020; Gu et al., 2022). Nevertheless, investigating the microbial diversity and composition within the BoB OMZs and their interactive relationships will help improve our understanding of ecosystem and biogeochemical cycling processes in this area.

In the present study, the microbial diversity and compositional structures and their interactive patterns were investigated between 90.04°E–97.26°E and 12.94°N–16.10°N, ranging from the Andaman Sea to the eastern BoB. High-throughput sequencing was used to evaluate the microbial composition of 131 water samples from the region. Further, the microbial diversity from oxygenic zones (OZs) to OMZs at depths of up to 200 m were characterized and their potential functions were evaluated.

## Materials and methods

### Sampling and geochemical analyses

Sampling was conducted at 23 stations across the Andaman Sea and the eastern BoB spanning water depths from 2 to 200 m through the international cooperation cruise, namely Joint Advanced Marine and Ecological Studies (JAMES) between China and Myanmar, during December 2019 to January 2020 (Supplementary Figure 1 and Supplementary Table 1). Seawater samples were collected with Niskin bottles mounted to a Sea-bird conductivity, temperature, and depth (CTD) sensor (SBE 911, Sea-Bird Co., WA, USA). A total of 2 L of seawater was filtered from each layer using 0.2-μm pore-size membrane filters (Millipore, Tullagreen, Carrigtwohill, Ireland). The collected filter samples were immediately frozen in liquid nitrogen and maintained at −20°C on board the ship. At the end of the cruise, the filters were moved to storage at −80°C in the lab until subsequent DNA extraction. Environmental parameters (i.e., depth, temperature, and salinity) were measured using a CTD sensor (SBE 911, Sea-Bird Co., WA, USA). Nutrient and DO levels were analyzed following previously described protocols (Grasshoff et al., 1999).

Oceanic OMZ region boundaries are fluid and their definitions have considerably varied, depending on oxygen concentration demands of marine organisms and varying thresholds for hypoxia among ocean regions (Karstensen et al., 2008; Vaquer-Sunyer and Duarte, 2008; Gilly et al., 2012, 2013). Further, the units used to define OMZ or hypoxic conditions are variable, and criteria have not been defined to identify these areas. In this study, the hypoxia threshold was defined as 0.7 mg/L and these samples were considered as OMZs, while mild hypoxia was defined at concentrations of 0.7–2 mg/L and considered as oxygen limited zones (OLZs), while samples with DO ≥ 2 mg/L were considered OZs (Supplementary Table 2). These definitions were made based on those of previous studies (Karstensen et al., 2008; Vaquer-Sunyer and Duarte, 2008; Gilly et al., 2012, 2013).

### Sequencing, data processing, and operational taxonomic unit assignments

DNA extraction and sequencing was conducted following previously described methods (Guo et al., 2020). The universal primer pair 338F (5′-ACTCCTACGGGAGGCAGCAG-3′) and 806R (5′ GGACTACHVGGGTWTCTAA T-3′) was used to amplify the V3–V4 hypervariable regions of 16S rRNA genes. Sequencing was subsequently conducted at Majorbio Bio-Pharm Technology Co. Ltd. (Shanghai, China). Raw sequences were processed using the QIIME pipeline (Caporaso et al., 2010). Raw data quality filtering and assembly were conducted using Fastp 0.19.6 and FLASH v1.2.11, respectively (Magoč and Salzberg, 2011; Chen et al., 2018). Raw reads were trimmed with a quality score threshold of tailing bases < 20 using moving-window sizes of 50 bp. If the average base pair quality in a window was < 20, sequences were trimmed and reads with length < 50 bp and those that contained ambiguous bases (N’s) were removed. Subsequently remaining paired-end reads were assembled with a minimum overlap length of 10 bp and with < 2% mismatches. Singletons were removed from the datasets and the remaining sequences were clustered into operational taxonomic units (OTUs) at a nucleotide similarity level of 97% using Uparse (version 7.0.1090) (Edgar, 2013). Taxonomic classification of each OTU was conducted using the RDP Classifier 2.11.^1^ Representative 16S rRNA gene sequences from each OTU were annotated against the SILVA database (silva132/16s_bacteria) using a similarity cutoff value of 0.7. The sequence information was listed in Supplementary Table 1. The raw sequence data generated in this study have been deposited in the NCBI Sequence Read Archive database under the accession number PRJNA862716.

### Data analysis

Alpha diversity indices including the Chao1 (richness), Heip (evenness), and Shannon (diversity) values were calculated using MOTHUR version 1.30.2 (Schloss et al., 2009). Mantel tests based on Bray–Curtis distances were used to assess the relationships of environmental factors and bacterial communities using the QIIME software package. Principal coordinates analysis (PCoA) was used to evaluate beta diversity patterns. To evaluate co-occurrence networks, the 500 most abundant OTUs were used to construct co-occurrence networks using Spearman’s correlation (r) relationships among abundances of OTUs. Correlations with *R* | ≥ | 0.8 and *p* < 0.01 were used for the final network analysis and visualized with the Gephi software program (version 0.9.2; WebAtlas, Paris, France). Module detection and topology parameter analysis was conducted in the Gephi 0.9.2 program. High degree, high closeness centrality, and low betweenness centrality values were used to define keystone taxa (Banerjee et al., 2018). The cutoff value for keystone taxa were 96, 0.477, 0.04 for degree, closeness centrality, and betweenness centrality, respectively. Random Forest analysis was used to identify significant indicator taxa associated with DO concentrations using the R randomForest package. LEfSe (Segata et al., 2011) analysis was used to identify microbial taxa that distinguished two or more groups using the all-against-all strategy and linear discriminant analysis (LDA) score thresholds of > 4. Heatmaps of environmental factors and important taxa were constructed from Spearman’s correlation analyses. Lastly, the functional annotation of prokaryotic taxa software program (FAPROTAX) was used to predict the potential functions of microbial communities. The important taxa were defined as the taxa that assigned to specific taxa, significantly different taxa, keystone taxa, and bioindicator taxa.

## Results

### Relationships between microbial communities and environmental factors

A total of 5,729 OTUs were generated from 16S rRNA gene sequencing analysis of 131 water samples. The Shannon alpha diversity index values of bacterial communities were negatively correlated with DO and temperature measurements (Table 1; Supplementary Figure 2), but positively correlated with depth, salinity, and NO_3_^–^ concentrations. The regression analysis *R*^2^ indicated that DO was the most correlated environmental factor to the Shannon index, while depth was the least correlated (Table 1). Mantel tests indicated that bacterial community composition was positively associated with environmental factors including DO, temperature, depth, salinity, and NO_3_^–^ concentrations (Table 2). DO exhibited the highest positive relationships with bacterial community composition. Thus, DO was the most important driver shaping the microbial communities in the OMZs of the Andaman Sea and eastern BoB above 200 m depth.

**TABLE 1:** Spearman’s correlation analysis between Shannon diversity and environmental factors.

Environmental factor	Correlation coefficient	Sig. (2-Tailed)	*R*^2^ of regression analysis	*P*
DO	−0.676	0.000	0.7343	0.000
Temperature	−0.731	0.000	0.5034	0.000
Depth	0.746	0.000	0.4245	0.000
Salinity	0.735	0.000	0.7188	0.000
NO_3_^–^	0.740	0.000	0.7135	0.000

**TABLE 2:** Mantel test results for environmental factors and bacterial community composition at the OTU level.

Environmental factor	*R*	*P*
DO	0.795	0.001
Temperature	0.687	0.001
Depth	0.681	0.001
Salinity	0.723	0.001
NO_3_^–^	0.702	0.001

### Bacterial community compositions and differences among oxygen zones

**FIGURE 1:**
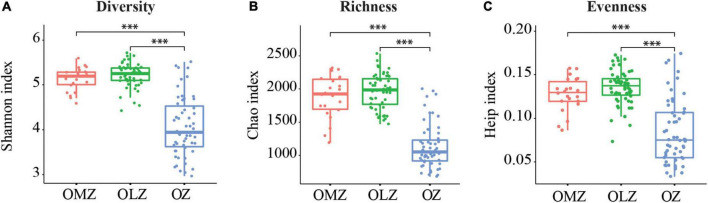
Distribution of alpha diversity index values for samples from the OMZ, OLZ, and OZ. **(A)** Diversity; **(B)** richness; and **(C)** evenness values are shown. Horizontal bars within boxes represent medians of each index. ^***^p < 0.001. Welch’s *t*-test was used in the statistical analysis.

**FIGURE 2:**
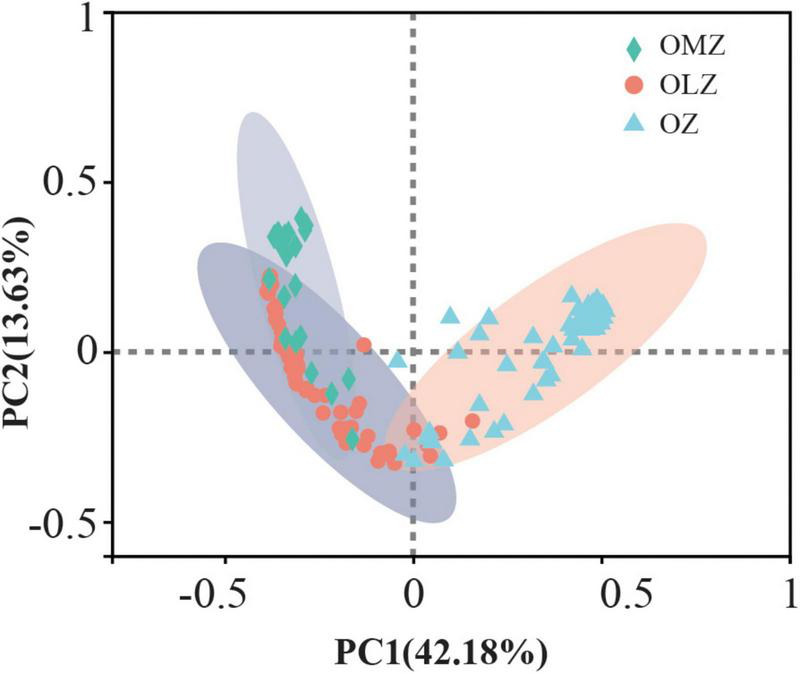
Principal coordinate analysis (PCoA) of Bray–Curtis distances among communities from the OMZ, OLZ, and OZ waters. ANOSIM was used in the statistical analysis.

Samples were separated into three groups based on DO concentrations including OMZs, OLZs, and OZs. The Shannon, Chao1, and Heip indices of bacterial community diversity were significantly higher in the OMZs and OLZs than in the OZs. Although these index values for OLZ communities were higher than in those of OMZ communities, significant differences were not detected (Figure 1). PCoA analysis (Figure 2, Bray–Curtis distance: *R* = 0.811, *p* = 0.001) was used to assess the distribution of microbial communities at the OTU level. The first PCoA axis explained 42.18% of the total variation and separated the OMZ and OZ communities. However, the microbial communities from OLZ could not be separated from the OMZ and OZ communities based on PCoA ordinations. PERMANOVA results (Supplementary Table 3) indicated that the DO showed the highest *R*^2^ (0.398) compared to other environmental factors. These results were corresponding to Mantel test result (Table 2), it indicated that DO was the most important environmental factors shaping the microbial communities in the Andaman Sea and eastern BoB above 200 m depth.

**FIGURE 3:**
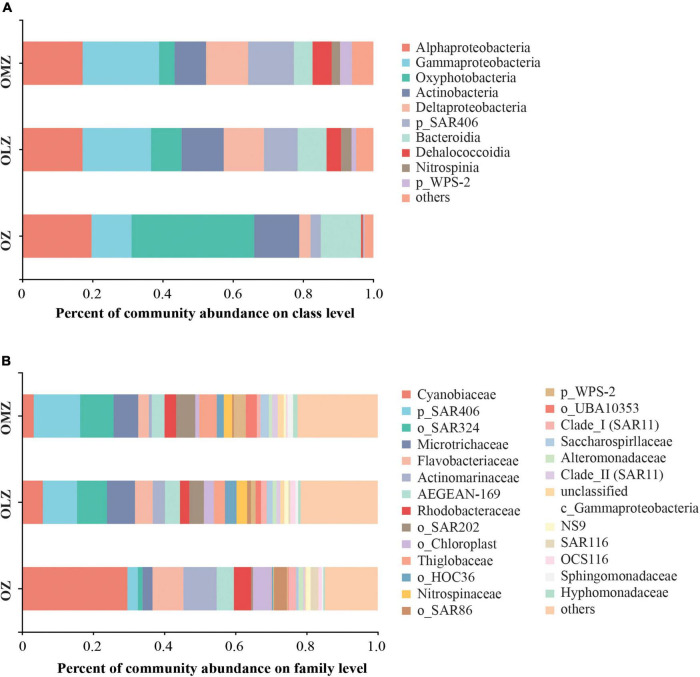
Microbial community composition of OMZ, OLZ, and OZ waters at the class and family levels. Taxa are distinguished at the **(A)** class and **(B)** family level. p, phylum; o, order; c, class.

A total of 79, 89, and 65 classes, in addition to 355, 391, and 312 families were identified in the OMZ, OLZ, and OZ communities, respectively (Supplementary Figure 3). Further, the predominant classes included the Oxyphotobacteria, Alphaproteobacteria, and Actinobacteria within the OZ communities, while the three most abundant classes of the OLZs were the Gammaproteobacteria, Alphaproteobacteria, and Actinobacteria, in addition to the SAR406 group in the OMZs. At the family level, the Cyanobiaceae, Flavobacteriaceae, and Actinomarinaceae dominated the OZ communities, while the SAR406, SAR324 marine group B, and Microtrichaceae families were most dominant in the OLZ and OMZ communities (Figure 3). The relative abundances of Alphaproteobacteria and Gammaproteobacteria were highest in the OMZ and lowest in the OZ (Figure 3). Moreover, the Deltaproteobacteria, SAR406, Dehalococcoidia, and WPS-2 exhibited gradually increased relative abundances with decreasing DO concentrations (from the OZ to the OMZ communities) (Figure 2).

A total of 16, 37, and 9 families were specific to the OMZ, OLZ, and OZ, communities, respectively (Supplementary Figure 3). The top three highest relative abundance specific taxa for the OMZ communities were Latescibacteraceae, MSBL5 (o), and Rhodobiaceae, while the most specific families for the OLZ communities were the Muribaculaceae, Thermodesulfovibrionia (c), and Rhizobiales (o). Lastly, the Bradymonadales (o), type III (Entomoplasmatales), and Pseudanabaenaceae were the top three highest relative abundance specific taxa for the OZ communities (Supplementary Table 4).

**FIGURE 4:**
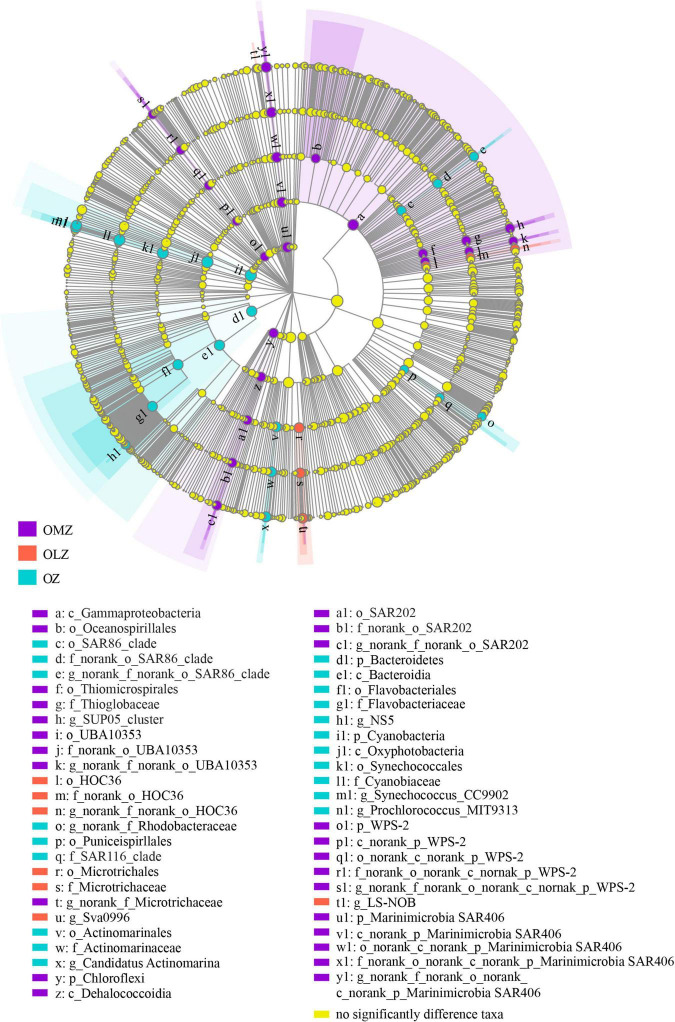
Taxonomic cladogram showing distinctive taxa in the OMZ, OLZ, and OZ communities identified by LEfSe analysis. Enlarged colored circles show differentially abundant taxa in each sampling zone. Yellow dots mean the taxa that no significant different between OMZ, OLZ, and OZ waters.

LEfSe analysis revealed significant differences of taxa among the OZ, OLZ, and OMZ communities. Specifically, the Thioglobaceae, SAR202, WPS-2 (p), UBA10353, and SAR406 were enriched in the OMZ communities, while the HOC36 and Microtrichaceae were enriched in the OLZ communities (Figure 4). The SAR86, SAR116, Actinomarinaceae, Flavobacteriaceae, and Cyanobiaceae groups were enriched in the OZ communities.

**FIGURE 5:**
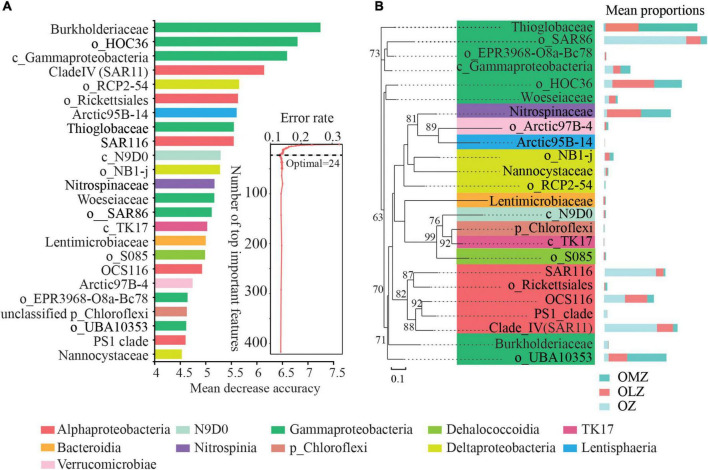
Random forest modeling analysis detection of microbial biomarker taxa that significantly differentiate OMZ, OLZ, and OZ communities. **(A)** The 24 most discriminatory microbial families were identified by applying Random Forest classification of the relative abundances of populations in the OMZ, OLZ, and OZ communities. Biomarker taxa are ranked in descending order of importance to the accuracy of the model. **(B)** Phylogenetic analysis of biomarker families. Bars represent the relative abundances of microbial taxa in the OMZ, OLZ, and OZ communities.

Random forest modeling was used to identify bioindicator taxa that differentiated OZ, OLZ, and OMZ communities at the family level (Figure 5A). Further, 10-fold cross-validation was used to evaluate the importance of bioindicator microbial families. The cross-validation error curve stabilized with an error rate of 0.129 when considering the 24 most relevant families. The 24 families were consequently identified as bioindicator taxa (Figure 5A). The families belong to 11 classes in addition to eight and five families, within the Gammaproteobacteria and Alphaproteobacteria, respectively (Figures 5A,B). Burkholderiaceae was the most important bioindicator taxa and their relative abundances were highest in the OZ communities (Figure 5B; Supplementary Figure 4). In addition, the relative abundances of SAR116, the SAR11 clade IV, and the PS1 clade were highest in the OZ. The HOC36, Nitrospinaceae, OCS116 (o), Woeseiaceae (o), NB1-j (o), Lentimicrobiaceae, RCP2-54, and N9D0 (c) groups exhibited the highest relative abundances in the OLZ (Figure 5B; Supplementary Figure 4). In addition, the Thioglobaceae, UBA10353 (o), Rickettsiales (o), and Chloroflexi (p) groups exhibited the highest relative abundances in the OMZ communities (Figure 5B; Supplementary Figure 4). The S085, TK17, and N9D0 (within the Chloroflexi phylum) were also identified as bioindicator taxa (Figure 5A). The relative abundances of these groups were very low, and they were more abundant in the OMZ and OLZ communities than in the OZ community (Figure 5B; Supplementary Figure 4). All of these taxa clustered into a single clade and exhibited close relationships with the Lentimicrobiaceae (Figure 5B). Unclassified HOC36 were the second most important bioindicator taxa (Figure 5A). OTUs affiliated with the HOC36 or *Candidatus* Thioglobus sp. taxa remain uncultured.

### Bacterial community co-occurrence patterns

**FIGURE 6:**
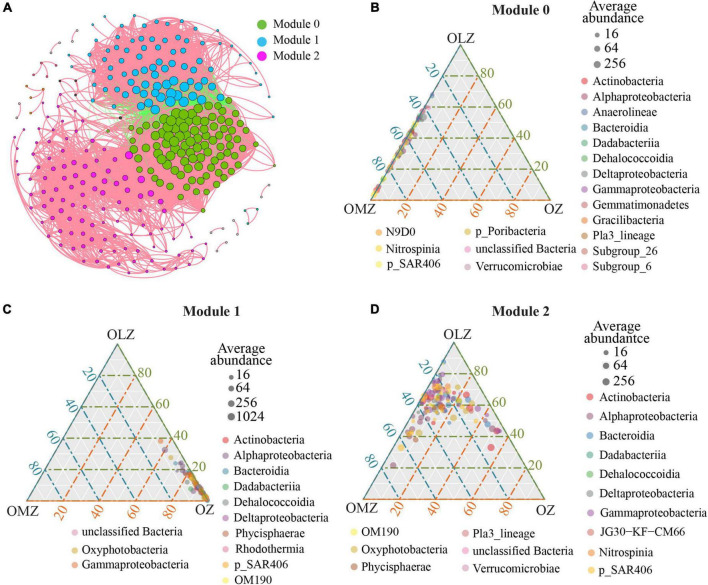
Co-occurrence patterns of microbial populations based on Spearman’s correlation analysis. **(A)** Co-occurrence network. Network nodes represent OTUs and the size of each node is proportional to the number of its associations (degree). Each node is colored in a module. A connection represents a strong and significant correlation of abundance (| r| > 0.8, *p* < 0.01). **(B)** Ternary plot showing the distribution of OTUs from module 0 at the class level. **(C)** Ternary plot showing the distribution of OTUs from module 1 at the class level. **(D)** Ternary plot showing the distribution of OTUs from module 2 at the class level. The gray dots indicate the average abundant of taxa.

A co-occurrence network was generated to explore bacterial interactions among bacterial communities (Figure 6A and Table 3). The network exhibited a scale-free degree distribution, suggesting non-random co-occurrence patterns (Supplementary Figure 5). The co-occurrence network comprised 324 nodes and 8,851 edges. A total of 7,191 positive correlations were present in the network in addition to 1,660 negative edges.

**TABLE 3:** Topological properties of community networks.

Network metric	Value
No. of nodes	324
No. of edges	8,851
Modularity (no of modules)	0.745 (14)
Average clustering coefficient	0.731
Network diameter	9
Average path length	2.61
Average degree	29.778
Density	0.169

The entire network was divided into 14 modules, with modules 0, 1, and 2 respectively accounting for 30.86, 26.23, and 33.95% of the entire network. Module 0 was associated with OMZ communities and predominantly comprised Dehalococcoidia, Gammaproteobacteria, and Alphaproteobacteria that constituted the largest relative abundances of 19.6, 18.6, and 16.5%, respectively, among module 0 (Figure 6B) nodes. Module 1 (Figure 6C) was associated with OZs, wherein Alphaproteobacteria, Bacteroidia, and Gammaproteobacteria contributed 29.4, 23.5, and 22.4% of the module 1 nodes, respectively. Lastly, module 2 (Figure 6D) was associated with OLZs. Alphaproteobacteria, Gammaproteobacteria, and SAR406 accounted for 21.2, 17.7, and 16.8% of the module 2 nodes, respectively.

Keystone taxa considerably influence community networks, in addition to maintaining their structures and functions (Berry and Widder, 2014; Banerjee et al., 2018). High degree, high closeness centrality, and low betweenness centrality values were used to identify keystone OTUs (Supplementary Table 5) that were affiliated with the SAR202, AEGEAN-169, UBA10353, SAR406, and Rhodobacteraceae groups, with most of these OTUs belonging to module 0. OTU6020 was affiliated with the Rhodobacteraceae and was a keystone taxa for module 1. In addition, OTU881 was affiliated with SAR406 and was a keystone taxa for module 2.

### Relationships between environmental factors and important taxa

**FIGURE 7:**
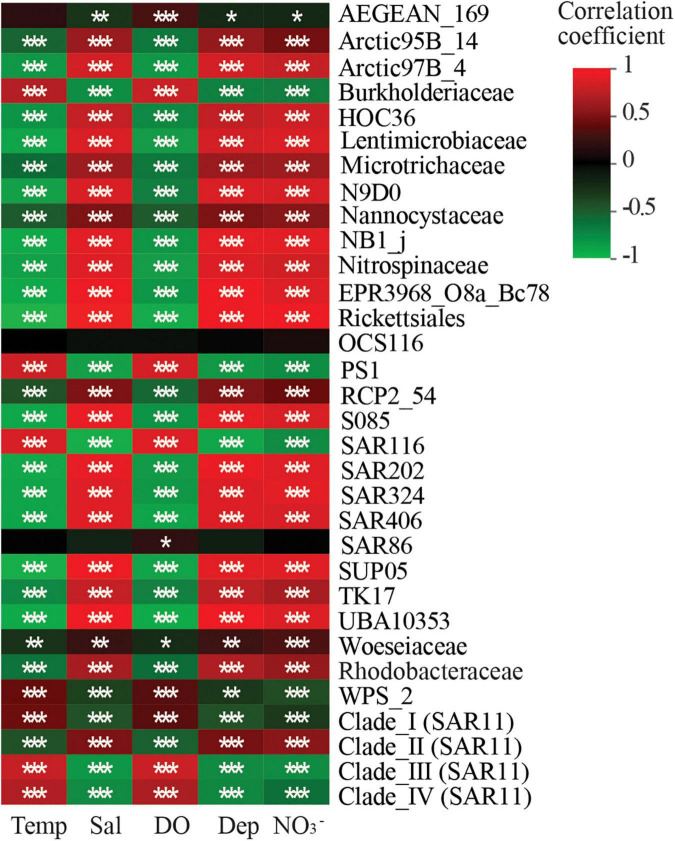
Heatmap showing the correlations of biomarker and keystone taxa with environmental factors. Temp, temperature; Sal, salinity; DO, dissolved oxygen; Dep, depth; NO_3_^–^, Spearman’s correlation analysis was employed in the correlation analysis of environmental factors and each taxon. Correlation coefficient value *R* were shown in different colors. *p < 0.05; ^**^p < 0.01; ^***^p < 0.001.

Spearman correlation analysis revealed that the relative abundances of most taxa were significantly correlated with measurements of DO, temperature, depth, salinity, and NO_3_^–^ concentrations (Figure 7). SAR86 relative abundances were only significantly positively correlated with DO (Figure 7; Supplementary Table 6). Correlations between AEGEAN-169 relative abundances and temperature were not observed. The relative abundances of Nitrospinaceae, SUP05, SAR406, SAR324, UBA10353, and HOC36 were negatively correlated with DO. Lastly, the relative abundances of Burkholderiaceae, PS1, Woeseiaceae, Clade I, Clade III, and Clade IV were positively correlated with DO.

### Functional predictions of bacterial communities

**FIGURE 8:**
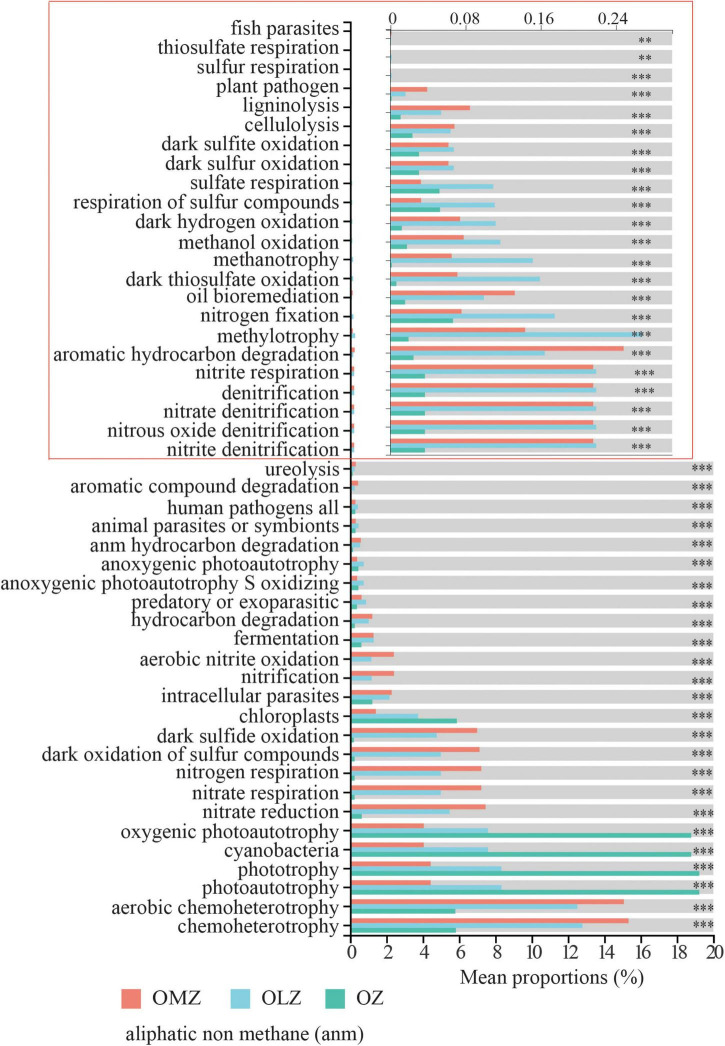
Functional Annotation of Prokaryotic Taxa (FAPROTAX) function predication plot showed mean proportions of predicated functions in OMZ, OLZ, and OZ. Games–Howell were used in the statistical test. ^**^p < 0.01; ^***^p < 0.001. Since the mean proportions of some functions (in red box) is very less, the x-axis was enlarged, and the number marked in the x-axis in red box was still the real mean proportions of each function.

Functional Annotation of Prokaryotic Taxa (FAPROTAX) analysis indicated the presence of various functional processes including chemoheterotrophy, nitrification, denitrification, and dark sulfite/sulfur oxidation that were more abundant in the OMZ and OLZ communities compared with the OZ communities (Figure 8). Denitrification potential was predicted for the communities, but the relative abundances were very low. In contrast, nitrification potential exhibited higher relative abundances than denitrification potential. Furthermore, the phototrophy and oxygenic photoautotrophy were more abundant in the OZ and OLZ compared to OMZ (Figure 8). FAPROTAX functional predictions for bioindicator taxa identified by random forest analysis suggested that bioindicator taxa for the OMZ and OLZ communities exhibited functions in nitrogen and sulfur cycling, including *via* nitrate reduction, nitrification, and dark sulfide/sulfur compound oxidation (Supplementary Figure 6). Among the indicator taxa for the OZ communities, aromatic hydrocarbon degradation, chemoheterotrophy, and ureolysis functions were prominent (Supplementary Figure 6).

## Discussion

The expansion and shoaling of OMZs have increased in recent decades and these processes remain ongoing. Microbial communities are important players in biogeochemical processes and feature prominently within OMZ generation and functioning (Bertagnolli and Stewart, 2018). In the present study, samples were collected from the epipelagic zone of the Andaman Sea and eastern BoB to assess the microbial ecology of waters exhibiting different oxygen concentrations across OMZs, OLZs, and OZs. The microbial community diversity, composition, bioindicator, and keystone taxa identified here suggest that major biogeochemical processes differ considerably between OZs and OMZs of the Andaman Sea and eastern BoB epipelagic waters.

### Environmental factors controlling the diversity and composition of microbial communities

Investigation of relationships between environmental factors with microbial community composition and diversity revealed that DO, depth, temperature, salinity, and NO_3_^–^ concentrations were the significant environment factors that structured microbial communities in the study region. These results indicated that DO, depth, temperature, salinity, and NO_3_^–^ should co-shape microbial communities. Among these, DO was the most highly associated environmental factor with microbial communities, and also significantly differentiated microbial community types. Thus, although microbial community structures were determined by multiple interacting environmental factors, DO was the most important driver of community structure in the epipelagic zone of the study site, likely due to the oxygen-depleted water column. DO has been similarly detected as an important and strong driver of microbial community diversity and community structure in hypoxic ocean regions like the Gulf of Mexico hypoxic zone and the eastern tropical North Pacific Ocean (ETNP) (Beman and Carolan, 2013; Campbell et al., 2018).

### Microbial community composition and important taxa in the Andaman Sea and eastern Bay of Bengal

Alpha diversity index comparisons suggested that microbial communities exhibited increased species richness and evenness in oxygen deficient (OMZ and OLZ) compared with oxygenic (OZ) water column communities. However, significant differences of diversity indices were not detected between OLZ and OMZ communities, indicating that the microbial community composition of OLZs was more similar to OMZ communities than OZ communities. PCoA also indicated that the microbial communities of OMZ and OZ samples were considerably different, while the microbial community compositions of OLZ overlapped with OZ and OMZ communities. OLZs are transition regions adjacent to OZs and OMZs, perhaps accounting for the mixed characteristics of the OLZ microbial communities. These results are consistent with those from the ETNP, wherein bacterial richness exhibited a unimodal distribution with decreasing DO, reaching maximum values at the edge of the OMZ, then decreasing (Beman and Carolan, 2013). These data indicate that the transition region from the OZ to OMZ contained greater types of microbiota (i.e., greater diversity).

The microbial communities analyzed in this study possessed characteristics common to other global OMZs. As previously shown (Wright et al., 2012; Long et al., 2021), the Oxyphotobacteria, Alphaproteobacteria, Actinobacteria, and SAR86 phyla are prevalent in oxygenic water columns overlying OMZs. Prevalent microbial taxa such as the Nitrospinaceae, SAR202, SAR406, SAR324, Thioglobaceae (primarily the SUP05 group), and UBA10353 previously observed in oxygen-deficient waters (Wright et al., 2012; Pajares et al., 2020; Long et al., 2021) were also identified in the OMZ/OLZ investigated in this study. Relatively high abundances of SAR202, SAR406, SAR324, SUP05, Nitrospinaceae, and UBA10353 have also been detected in many OMZs, including those in the ETNP, Northeast subarctic Pacific Ocean, Arabian Sea, and Black Sea (Fuchsman et al., 2011; Allers et al., 2013; Beman and Carolan, 2013; Lüke et al., 2016; Thrash et al., 2017; Pajares et al., 2020). The family affiliated with the SAR324 was the second most predominant taxa in the OMZ of this study, while the SAR202, SAR406, and UBA10353 groups were not only abundant, but also keystone taxa that maintained the network structure of interactions among the OMZ/OLZ communities of the Andaman Sea and eastern BoB. The SAR324, SAR202, and SAR406 groups ubiquitously inhabit a wide variety of environments, and their abundances are higher in deeper waters or in low-oxygen concentration waters such as OMZs (Giovannoni et al., 1996; Morris et al., 2004; Wright et al., 2012, 2014; Rinke et al., 2013; Sheik et al., 2014; Guerrero-Feijóo et al., 2018). SAR324 possess metabolic flexibility allowing their use of several electron donors including sulfur, hydrocarbons, C1 compounds, and organic carbon, in addition to the use of several electron acceptors such as nitrite or oxygen (Sheik et al., 2014). SAR202 members possess versatile metabolic functions including nitrate/nitrous oxide reduction in addition to the metabolism of complex carbohydrates and organosulfur compounds (Thrash et al., 2017; Mehrshad et al., 2018). Likewise, SAR406 genomes encode genes involved in dissimilatory sulfur oxidation and reduction, dissimilatory nitrite reduction to ammonia, and degradation of complex carbohydrate compounds (Wright et al., 2014; Thrash et al., 2017). Nevertheless, the SAR202 and SAR406 groups contain multiple sub-lineages that may possess distinct metabolic functions in various environmental conditions (Thrash et al., 2017). Moreover, the gene expression of certain nitrogen/sulfur cycling genes were closely correlated to DO concentrations (Thrash et al., 2017).

UBA10353 and AEGEAN-169 were also keystone taxa in the network analysis. Unlike the SAR202 and SAR406 groups, the ecology, genetics, and functions of UBA10353 and AEGEAN-169 have not been well-documented. Indeed, the identification of UBA10353 has only been reported in recent years. However, more abundant populations have been identified in deeper layers of the western Mediterranean Sea and the OMZ core of Tropical Mexican Pacific, and they also have been shown to exhibit the potential ability for carbon fixation and sulfur oxidation (Pajares et al., 2020; Martínez-Pérez et al., 2022; Mena et al., 2022).

The relative abundances of AEGEAN-169 were similar between OMZ, OLZ, and OZ communities, but still exhibited the highest relative abundances in OZ samples of this study. The AEGEAN-169 group is closely related to SAR11 (Alonso-Sáez et al., 2007), and is generally present throughout water columns (Cram et al., 2015a; Reintjes et al., 2019). The group is also particularly abundant in the surface waters of the Ultraoligotrophic South Pacific Gyre and most abundant at 500 m within the San Pedro Channel (Cram et al., 2015a; Reintjes et al., 2019). Several environmental factors, such as oxygen, salinity, NO_3_^–^, influence the distribution of AEGEAN-169 (Cram et al., 2015b). The correlation of oxygen concentration with AEGEN-169 were different among OTUs, and both negative and positive relationship have been detected (Cram et al., 2015b). The different adaption characteristic for oxygen of AEGEAN-169 ecotypes might contribute to the similar relative abundance between OMZ, OLZ, and OZ communities.

Arctic97B-4 (affiliated with Verrucomicrobia) was identified as a bioindicator taxa in this study and have also been detected in other oxygen-deficient waters, including the OMZs of the Arabian Sea and Cariaco Basin (Lüke et al., 2016; Suter et al., 2018). The ecological roles of Arctic97B-4 remain unclear, but Verrucomicrobia taxa might be capable of surviving under oxygen-depleted conditions and potentially oxidizing methanol or methane (Dalcin Martins et al., 2021). A strong association between Arctic97B-4 and SAR202 was also detected and both were simultaneously present in modules 1 and 2 (Supplementary Figure 7). Tight associations of SAR202 and Arctic97B-4 have been detected in coastal waters that are also oxygen deficient water environments (Suter et al., 2018; Chun et al., 2021). Arctic97B-4, SAR202, and SAR406 taxa have been repeatedly shown to be associated in modules related to nutrient compositions, with the taxa exhibiting functions related to nitrate reduction, nitrification, and dark sulfide oxidation (Chun et al., 2021). Arctic97B-4 taxa exhibit unclear functions, but frequently share distribution patterns with SAR202 and SAR406 taxa, implying they share a similar functional role.

The Nitrospinaceae (including *Nitrospina* and *LS-NOB*) and SUP05 groups were significantly abundant in the OLZ and OMZ communities of this study and were also important indicator taxa. *Nitrospina* are aerobes and major nitrite oxidizing bacteria that contribute to nitrification in marine environments (Sun et al., 2019; Beman et al., 2021). Their members are often detected in global OMZs, as in OMZs of the Arabian Sea, ETSP, ETNP, and BoB (Sun et al., 2019; Gu et al., 2022). The relatively higher abundances of *Nitrospina* in the OLZ indicate that they might contribute oxygen-depletion and nitrification functions in the Andaman Sea and eastern BoB. SUP05 is also highly associated with globally distributed OMZs. The group includes known chemolithoautotrophs that have the potential capacity for carbon fixation, sulfur oxidation, and nitrate/nitrite reduction in oxygen-deficient oceanic waters (Walsh et al., 2009; Shah et al., 2017; Mattes et al., 2021). The HOC36 group (including uncultured taxa and *Candidatus* Thioglobus sp.) were the second most important bioindicator taxa. *Candidatus* Thioglobus sp. belongs to the SUP05 clade (Marshall and Morris, 2013), suggesting that HOC36 might exhibit similar functions as SUP05 (Thioglobaceae). These data suggest that the SAR324, SAR202, SAR406, Nitrospinaceae, SUP05, and UBA10353 taxa could contribute to carbon, nitrogen, or sulfur cycling alongside oxygen depletion in the OMZ and OLZ of the Andaman Sea and eastern BoB epipelagic waters.

Several taxa not previously reported extensively in OMZs, including the WPS-2, Microtrichaceae, and Woeseiaceae, were identified as bioindicator taxa in this study. In particular, the WPS-2 and Microtrichaceae were especially abundant in the OMZ and OLZ communities of this study. WPS-2 exhibits a global distribution and is most often found in cool, acidic, and aerobic environments, contributing to their hypothesized functions as aerobic or microaerobic taxa (Ward et al., 2019) that may also perform chemolithoautotrophy (Sheremet et al., 2020; Ji et al., 2021). However, the WPS-2 have rarely been identified in marine environments. Thus, the identification of higher relative abundances of WPS-2 in OLZs or OMZs might imply that WPS-2 contribute to oxygen depletion in this region. Microtrichaceae have been shown to play roles in nitrification-anammox systems and can hydrolyze and metabolize complex organic matter (Wang et al., 2020; Li et al., 2021). Woeseiaceae is one of the most abundant bacterial families in marine sediments (Mußmann et al., 2017; Moreno-Ulloa et al., 2020; Zhou et al., 2022), and might contribute to sulfur-oxidation (Dyksma et al., 2016; Mußmann et al., 2017). In addition, RCP2-54 might be involved in methane, sulfur, and nitrogen cycling activities in Barents Sea sediments (Begmatov et al., 2021). These data suggest that most of the bioindicator taxa with higher relative abundances in the OMZ and OLZ communities of this study exhibit wide capacities for oxygen depletion, sulfur cycling, and nitrogen cycling.

Burkholderiaceae, Clade IV, SAR116, and SAR86 taxa were also identified as bioindicator taxa that were abundant in the OZ. Burkholderiaceae was the most significant bioindicator taxa identified in this study. Burkholderiaceae are associated with organic contaminants and have been detected in greater abundance in coastal shallow ecosystems such as in the Baltic Sea and south China sea, where they have been partially affected by anthropogenic activities (Iburg et al., 2021; Zhang et al., 2021). Some of the sampling locations in this study included continental shelf seas and near coastal estuaries, which could be impacted by pollutants, leading to anthropogenic influences. Clade IV belongs to the SAR11 group and was the fourth most important bioindicator taxa. SAR11 are prevalent in oxygen-rich surface oceans and are also abundant in OMZs (Wright et al., 2012; Tsementzi et al., 2016). SAR11 members can respond to organic matter produced autochthonously by phytoplankton release or *via* food web production of dissolved organic carbon (Schwalbach et al., 2010; Morris et al., 2012), while possessing the ability to reduce nitrate in anoxic zones (Tsementzi et al., 2016). The group has also been identified as abundant taxa that might be important nitrate reducers in the BoB OMZ (Gu et al., 2022). The SAR11 group contains several subclades including Clade I and Clade II that were also identified in samples of this study, to the exclusion of Clade IV. Similar to Clade IV, Clade I is also more abundant in OZ samples, while Clade II exhibited increased relative abundance with decreasing DO concentrations. Adverse effects from environmental factors on the abundances of Clade I and IV with Clade II were also detected in this study (Figure 7). These data suggest that different subclades of SAR11 might differentially contribute to oxygenic zone communities *via* different functions, while also contributing to nitrogen cycling in the Andaman Sea and eastern BoB. SAR86 and SAR116 are ubiquitous and abundant taxa that also differentiated communities in the Andaman Sea and BoB. The functions and ecological characteristics of different SAR86 ecotypes might shape their variable distribution patterns, while the SAR86 are most abundant in surface oceans and are also prevalent in oxygenic waters overlying OMZs (Reintjes et al., 2019; Hoarfrost et al., 2020; Mena et al., 2020). SAR116 exhibited decreased OTU numbers in the OMZ of the ETNP in association with decreasing DO levels (Beman and Carolan, 2013), indicating decreasing ecotype prevalence with decreasing oxygen concentrations. The positive correlation between the relative abundances of SAR116 and SAR86 indicate that they prefer the oxygenic waters of the Andaman Sea and eastern BoB. Cyanobiaceae is one of family that showed highest relative abundance in the OZ, and the relative high abundance were also detected in the OLZ and OMZ waters. These groups should contribute to autotrophy/oxygenic photoautotrophy of predicated function. The cyanobacteria such as *Prochlorococcus* and *Synechococcus* are commonly present with high abundance in the euphotic waters of OMZ regions (Beman and Carolan, 2013; Gu et al., 2022). The activity of cyanobacteria is largely light dependent that could contribute to large percentage of global primary production (Flombaum et al., 2013). However, the low-light clade of *Prochlorococcus* has been detected in ETSP and Arabian Sea, the OMZ shoaling into the euphotic zone may expand the niche of these type cyanobacteria (Goericke et al., 2000; Lavin et al., 2010; Gilly et al., 2013).

### Potential biogeochemical cycling functions

Functional prediction revealed potential biogeochemical cycling activities among the taxa in the Andaman Sea and eastern BoB. Functions including nitrate/nitrite respiration/reduction, nitrification, denitrification, chemoheterotrophy, and hydrocarbon degradation exhibited increased predicted proportions in the OMZ and OLZ water samples of the Andaman Sea and eastern BoB. Nitrogen and sulfur metabolisms are important for nutrient cycling in OMZs (Wright et al., 2012; Long et al., 2021). Nitrogen cycling activities including nitrification and denitrification are extensively present in oxygen-deficient waters such as in OMZs, and OMZs might account for 30–50% of oceanic nitrogen loss (Lam and Kuypers, 2011; Kuypers et al., 2018). In the BoB, low oxygen concentrations could support denitrifier and anammox microbial populations that mediate low, but significant N loss (Bristow et al., 2016). Although potential microbial populations that could be denitrifiers were identified here, the actual *in situ* metabolisms of these populations might closely depend on oxygen concentrations (Lam and Kuypers, 2011; Bristow et al., 2016; Gu et al., 2022). Oxygen concentrations within all samples from the OMZ in this study ranged from 0.27 to 0.7 mg L^–1^. Within samples exhibiting these concentrations, some microbial groups (e.g., SAR11) encoding genes involved in nitrification exhibited higher levels in the BoB OMZ (Gu et al., 2022). In the present study, predicted denitrification gene abundances were considerably lower than nitrification gene abundances. These data imply that nitrification and denitrification activities might co-exist and that nitrification is a potential major nitrogen cycling activity in the OMZ region of the Andaman Sea and the eastern BoB.

Sulfur cycling–related functional genes such as those involved in dark sulfide/sulfur compound, sulfur, and sulfite oxidation also exhibited higher predicted abundances in OMZ/OLZ water samples than in oxygenic water samples. The OMZ sulfur cycle comprises abiotic and biologically mediated reactions (van Vliet et al., 2021). However, it is difficult to investigate sulfur reactions *in situ*, with the exception sulfide oxidation and sulfate reduction, due to technological difficulties (van Vliet et al., 2021). Nevertheless, investigations of sulfur-based microorganisms have greatly improved our understanding of sulfur cycling in oceanic OMZs (Callbeck et al., 2021; van Vliet et al., 2021). Microorganisms potentially involved in sulfur cycling including the SUP05, SAR324, and SAR406 were detected in the Andaman Sea and eastern BoB communities of the present study. These sulfur cycle–associated bacterial taxa might also potentially couple nitrogen and carbon cycling (Mußmann et al., 2017; Thrash et al., 2017; Callbeck et al., 2018; van Vliet et al., 2021).

Microbial populations that mediate nitrogen and sulfur cycling or other processes such as aerobic chemoheterotrophy also exhibit aerobic and/or anaerobic metabolisms that can coexist and reach stable equilibrium to a certain extent in anoxic marine zones (Zakem et al., 2020). Aerobic metabolisms such as aerobic chemoheterotrophy can consume oxygen in the water column. Thus, microbial communities, and especially bioindicator and keystone taxa identified here, including SAR406, SAR202, Thioglobaceae, and Nitrospinaceae, mediate nitrogen or sulfur cycling and potentially account for oxygen consumption in the OMZ of the Andaman Sea and eastern BoB.

## Conclusion

The microbial community profiles of the Andaman Sea and eastern BoB epipelagic waters including oxygenic and oxygen-deficient samples were characterized here. Microbial community diversity and composition were highly correlated with environmental factors including DO, pH, temperature, salinity, and NO_3_^–^ concentrations. DO was the most highly correlated environmental factor with microbial community composition. Microbial diversity, richness, and evenness were highest in the OLZ and lowest in the OZ, while microbial composition was significantly different between the OMZs and OZs of epipelagic waters. LEfSe, random forest modeling, and network analysis revealed significant differences of taxa, bioindicators, and keystone taxa within the OMZ, OLZ, and OZ environments. Taxa such as SUP05, SAR202, SAR406, WPS-2, and UBA10353 were abundant in the OMZ, while SAR202, SAR406, and UBA10353 were also keystone taxa in the microbial interaction network. A total of 24 taxa, including the Burkholderiaceae, HOC36, SAR11 Clade IV, and Nitrospinaceae groups, were identified as bioindicator taxa that differentiated OMZ, OLZ, and OZ communities of the Andaman Sea and eastern BoB epipelagic waters. Furthermore, functional prediction analysis indicated that nitrogen and sulfur cycling taxa potentially increased with decreased DO levels. Several microbial groups, including Nitrospinaceae, WPS-2, SAR324, SAR202, SAR406, SUP05, and UBA10353, might contribute to nitrogen and sulfur cycling, in addition to oxygen consumption in these areas. These results indicate that the core OMZ communities are generally distributed in much deeper waters, microbial community composition and biogeochemical cycling activities of OMZs are significantly different from those in the oxygenic, epipelagic waters.

## Data availability statement

The datasets presented in this study can be found in online repositories. The names of the repository/repositories and accession number(s) can be found in the article/Supplementary material.

## Author contributions

RG conceived the study, carried out the sampling and experiments, conducted data analyses, and wrote the manuscript. XM, HL, and JZ carried out the sampling and conducted data analysis. CT, TW, NA, HW, and CL carried out the sampling. SN revised the manuscript. FZ and PW conceived the study and reviewed and revised the manuscript. All authors reviewed the manuscript and approved the submitted version.

## Footnotes

1
https://sourceforge.net/projects/rdp-classifier/

## References

[B1] Allers E. Wright J. J. Konwar K. M. Howes C. G. Beneze E. Hallam S. J. (2013). Diversity and population structure of Marine Group A bacteria in the Northeast subarctic Pacific Ocean. *ISME J.* 7 256–268. 10.1038/ismej.2012.108 23151638 PMC3554399

[B2] Alonso-Sáez L. Balagué V. Sà E. L. Sánchez O. González J. M. Pinhassi J. (2007). Seasonality in bacterial diversity in north-west Mediterranean coastal waters: assessment through clone libraries, fingerprinting and FISH. *FEMS Microbiol. Ecol.* 60 98–112. 10.1111/j.1574-6941.2006.00276.x 17250750

[B3] Banerjee S. Schlaeppi K. van der Heijden M. G. A. (2018). Keystone taxa as drivers of microbiome structure and functioning. *Nat. Rev. Microbiol.* 16 567–576. 10.1038/s41579-018-0024-1 29789680

[B4] Begmatov S. Savvichev A. S. Kadnikov V. V. Beletsky A. V. Rusanov I. I. Klyuvitkin A. A. (2021). Microbial communities involved in methane, sulfur, and nitrogen cycling in the Sediments of the Barents Sea. *Microorganisms* 9:2362. 10.3390/microorganisms9112362 34835487 PMC8625253

[B5] Beman J. M. Carolan M. T. (2013). Deoxygenation alters bacterial diversity and community composition in the ocean’s largest oxygen minimum zone. *Nat. Commun.* 4:2705. 10.1038/ncomms3705 24162368

[B6] Beman J. M. Vargas S. M. Wilson J. M. Perez-Coronel E. Karolewski J. S. Vazquez S. (2021). Substantial oxygen consumption by aerobic nitrite oxidation in oceanic oxygen minimum zones. *Nat. Commun.* 12:7043. 10.1038/s41467-021-27381-7 34857761 PMC8639706

[B7] Berry D. Widder S. (2014). Deciphering microbial interactions and detecting keystone species with co-occurrence networks. *Front. Microbiol.* 5:219. 10.3389/fmicb.2014.00219 24904535 PMC4033041

[B8] Bertagnolli A. D. Stewart F. J. (2018). Microbial niches in marine oxygen minimum zones. *Nat. Rev. Microbiol.* 16 723–729. 10.1038/s41579-018-0087-z 30250271

[B9] Breitburg D. Levin L. A. Oschlies A. Grégoire M. Chavez F. P. Conley D. J. (2018). Declining oxygen in the global ocean and coastal waters. *Science* 359:eaam7240. 10.1126/science.aam7240 29301986

[B10] Bristow L. A. Callbeck C. M. Larsen M. Altabet M. A. Dekaezemacker J. Forth M. (2016). N2 production rates limited by nitrite availability in the Bay of Bengal oxygen minimum zone. *Nat. Geosci.* 10 24–29. 10.1038/NGEO2847

[B11] Bush T. Diao M. Allen R. J. Sinnige R. Muyzer G. Huisman J. (2017). Oxic-anoxic regime shifts mediated by feedbacks between biogeochemical processes and microbial community dynamics. *Nat. Commun.* 8:789. 10.1038/s41467-017-00912-x 28986518 PMC5630580

[B12] Callbeck C. M. Canfield D. E. Kuypers M. Yilmaz P. Bristow L. A. (2021). Sulfur cycling in oceanic oxygen minimum zones. *Limnol. Oceanogr.* 66 2360–2392. 10.1002/lno.11759

[B13] Callbeck C. M. Lavik G. Ferdelman T. G. Fuchs B. Gruber-Vodicka H. R. Hach P. F. (2018). Oxygen minimum zone cryptic sulfur cycling sustained by offshore transport of key sulfur oxidizing bacteria. *Nat. Commun.* 9:1729. 10.1038/s41467-018-04041-x 29712903 PMC5928099

[B14] Campbell L. G. Thrash C. Rabalais N. N. Mason O. U. (2018). Extent of the annual Gulf of Mexico hypoxic zone influences microbial community structure. *PLoS One* 14:e0209055. 10.1371/journal.pone.0209055 31022199 PMC6483191

[B15] Caporaso J. G. Kuczynski J. Stombaugh J. Bittinger K. Bushman F. D. Costello E. K. (2010). QIIME allows analysis of high-throughput community sequencing data. *Nat. Methods* 7 335–336. 10.1038/nmeth.f.303 20383131 PMC3156573

[B16] Chen S. Zhou Y. Chen Y. Gu J. (2018). fastp: an ultra-fast all-in-one FASTQ preprocessor. *Bioinformatics* 34 i884–i890. 10.1093/bioinformatics/bty560 30423086 PMC6129281

[B17] Chun S.-J. Cui Y. Baek S. H. Ahn C.-Y. Oh H.-M. (2021). Seasonal succession of microbes in different size-fractions and their modular structures determined by both macro- and micro-environmental filtering in dynamic coastal waters. *Sci. Total. Environ.* 784:147046. 10.1016/j.scitotenv.2021.147046 33894601

[B18] Cram J. A. Chow C. E. Sachdeva R. Needham D. M. Parada A. E. Steele J. A. (2015a). Seasonal and interannual variability of the marine bacterioplankton community throughout the water column over ten years. *ISME J.* 9 563–580. 10.1038/ismej.2014.153 25203836 PMC4331575

[B19] Cram J. A. Xia L. C. Needham D. M. Sachdeva R. Sun F. Fuhrman J. A. (2015b). Cross-depth analysis of marine bacterial networks suggests downward propagation of temporal changes. *ISME J.* 9 2573–2586. 10.1038/ismej.2015.76 25989373 PMC4817623

[B20] Dalcin Martins P. de Jong A. Lenstra W. K. van Helmond N. Slomp C. P. Jetten M. S. M. (2021). Enrichment of novel Verrucomicrobia, Bacteroidetes, and Krumholzibacteria in an oxygen-limited methane- and iron-fed bioreactor inoculated with Bothnian Sea sediments. *MicrobiologyOpen* 10:e1175. 10.1002/mbo3.1175 33650794 PMC7914226

[B21] Deutsch C. Brix H. Lto T. Frenzel H. Thompson L. A. (2011). Climate-Forced variability of ocean hypoxia. *Science* 333 336–339. 10.1126/science.1202422 21659566

[B22] Diaz R. Rosenberg R. (2008). Spreading dead zones and consequences for marine ecosystems. *Science* 321 926–929. 10.1126/science.1156401 18703733

[B23] Dutta K. Bhushan R. Somayajulu B. L. (2007). Rapid vertical mixing rates in deep waters of the Andaman Basin. *Sci. Total. Environ.* 384 401–408. 10.1016/j.scitotenv.2007.04.041 17586000

[B24] Dyksma S. Bischof K. Fuchs B. M. Hoffmann K. Meier D. Meyerdierks A. (2016). Ubiquitous Gammaproteobacteria dominate dark carbon fixation in coastal sediments. *ISME J.* 10 1939–1953. 10.1038/ismej.2015.257 26872043 PMC4872838

[B25] Edgar R. C. (2013). UPARSE: highly accurate OTU sequences from microbial amplicon reads. *Nat. Methods* 10 996–998. 10.1038/nmeth.2604 23955772

[B26] Fernandes G. L. Shenoy B. D. Damare S. R. (2020). Diversity of bacterial community in the oxygen minimum zones of Arabian Sea and Bay of Bengal as deduced by illumina sequencing. *Front. Microbiol.* 10:3153. 10.3389/fmicb.2019.03153 32038585 PMC6985565

[B27] Flombaum P. Gallegos J. L. Gordillo R. A. Rincón J. Zabala L. L. Jiao N. (2013). Present and future global distributions of the marine Cyanobacteria *Prochlorococcus* and *Synechococcus*. *Proc. Natl. Acad. Sci. U.S.A.* 110 9824–9829. 10.1073/pnas.1307701110 23703908 PMC3683724

[B28] Fuchsman C. A. Kirkpatrick J. B. Brazelton W. J. Murray J. W. Staley J. T. (2011). Metabolic strategies of free-living and aggregate-associated bacterial communities inferred from biologic and chemical profiles in the Black Sea suboxic zone. *FEMS Microbiol. Ecol.* 78 586–603. 10.1111/j.1574-6941.2011.01189.x 22066565

[B29] Gilly W. F. Beman J. M. Litvin S. Y. Robison B. H. (2013). Oceanographic and biological effects of shoaling of the oxygen minimum zone. *Ann. Rev. Mar. Sci.* 5 393–420. 10.1146/annurev-marine-120710-100849 22809177

[B30] Gilly W. F. Zeidberg L. D. Booth J. Stewart J. S. Marshall G. Abernathy K. (2012). Locomotion and behavior of Humboldt squid, *Dosidicus gigas*, in relation to natural hypoxia in the Gulf of California, Mexico. *J. Exp. Biol.* 215 3175–3190. 10.1242/jeb.072538 22915711

[B31] Giovannoni S. J. Rappé M. S. Vergin K. L. Adair N. L. (1996). 16S rRNA genes reveal stratified open ocean bacterioplankton populations related to the Green Non-Sulfur bacteria. *Proc. Natl. Acad. Sci. U.S.A.* 93 7979–7984. 10.1073/pnas.93.15.7979 8755588 PMC38860

[B32] Goericke R. Olson R. J. Shalapyonok A. (2000). A novel niche for *Prochlorococcus* sp. in low-light suboxic environments in the Arabian Sea and the Eastern Tropical North Pacific. *Deep Sea Res. I Oceanogr. Res. Pap.* 47 1183–1205. 10.1016/S0967-0637(99)00108-9

[B33] Grasshoff K. Kremling K. Ehrhardt M. (1999). *Methods of seawater analysis.* Weinheim: Verlag Chemie Press.

[B34] Gu B. Liu J. Cheung S. Ho N. H. E. Tan Y. Xia X. (2022). Insights into prokaryotic community and its potential functions in nitrogen metabolism in the Bay of Bengal, a pronounced Oxygen Minimum Zone. *Microbiol. Spectr.* 10:e00892-21. 10.1128/spectrum.00892-21 35579458 PMC9241787

[B35] Guerrero-Feijóo E. Sintes E. Herndl G. J. Varela M. M. (2018). High dark inorganic carbon fixation rates by specific microbial groups in the Atlantic off the Galician coast (NW Iberian margin). *Environ. Microbiol.* 20 602–611. 10.1111/1462-2920.13984 29124858

[B36] Guo R. Wang P. Lu D. Dai X. (2020). Comparison of bacterial communities associated with *Prorocentrum donghaiense* and *Karenia mikimotoi* strains from Chinese coastal waters. *Mar. Freshw. Res.* 71 1662–1671. 10.1071/MF20035

[B37] Han W. Mccreary J. P. (2001). Modeling salinity distributions in the Indian Ocean. *J. Geophys. Res.* 106 859–877. 10.1029/2000JC000316

[B38] Hoarfrost A. Nayfach S. Ladau J. Yooseph S. Arnosti C. Dupont C. L. (2020). Global ecotypes in the ubiquitous marine clade SAR86. *ISME J.* 14 178–188. 10.1038/s41396-019-0516-7 31611653 PMC6908720

[B39] Iburg S. Izabel-Shen D. Austin ÅN. Hansen J. P. Eklöf J. S. Nascimento F. J. A. (2021). Effects of recreational boating on microbial and meiofauna diversity in coastal shallow ecosystems of the Baltic Sea. *mSphere* 6:e00127-21. 10.1128/mSphere.00127-21 34468165 PMC8550262

[B40] Ji M. Williams T. J. Montgomery K. Wong H. L. Zaugg J. Berengut J. F. (2021). *Candidatus* Eremiobacterota, a metabolically and phylogenetically diverse terrestrial phylum with acid-tolerant adaptations. *ISME J.* 15 2692–2707. 10.1038/s41396-021-00944-8 33753881 PMC8397712

[B41] Jithin A. K. Francis P. A. (2020). Role of internal tide mixing in keeping the deep Andaman Sea warmer than the Bay of Bengal. *Sci. Rep.* 10:11982. 10.1038/s41598-020-68708-6 32686742 PMC7371704

[B42] Karstensen J. Stramma L. Visbeck M. (2008). Oxygen minimum zones in the eastern tropical Atlantic and Pacific oceans. *Prog. Oceanogr.* 77 331–350. 10.1016/j.pocean.2007.05.009

[B43] Kuypers M. M. M. Marchant H. K. Kartal B. (2018). The microbial nitrogen-cycling network. *Nat. Rev. Microbiol.* 16 263–276. 10.1038/nrmicro.2018.9 29398704

[B44] Lam P. Kuypers M. M. (2011). Microbial nitrogen cycling processes in oxygen minimum zones. *Ann. Rev. Mar. Sci.* 3 317–345. 10.1146/annurev-marine-120709-142814 21329208

[B45] Lavin P. González B. Santibáñez J. F. Scanlan D. J. Ulloa O. (2010). Novel lineages of *Prochlorococcus* thrive within the oxygen minimum zone of the eastern tropical South Pacific. *Environ. Microbiol. Rep.* 2 728–738. 10.1111/j.1758-2229.2010.00167.x 23766277

[B46] Levin L. A. (2018). Manifestation, drivers, and emergence of open ocean deoxygenation. *Ann. Rev. Mar. Sci.* 10 229–260. 10.1146/annurev-marine-121916-063359 28961073

[B47] Li J. Zheng L. Ye C. Ni B. Wang X. Liu H. (2021). Evaluation of an intermittent-aeration constructed wetland for removing residual organics and nutrients from secondary effluent: performance and microbial analysis. *Bioresour. Technol.* 329:124897. 10.1016/j.biortech.2021.124897 33657501

[B48] Long A. M. Jurgensen S. K. Petchel A. R. Savoie E. R. Brum J. R. (2021). Microbial ecology of oxygen minimum zones Amidst Ocean deoxygenation. *Front. Microbiol.* 12:748961. 10.3389/fmicb.2021.748961 34777296 PMC8578717

[B49] Lüke C. Speth D. R. Kox M. Villanueva L. Jetten M. (2016). Metagenomic analysis of nitrogen and methane cycling in the Arabian Sea oxygen minimum zone. *PeerJ* 4:e1924. 10.7717/peerj.1924 27077014 PMC4830246

[B50] Magoč T. Salzberg S. L. (2011). FLASH: fast length adjustment of short reads to improve genome assemblies. *Bioinformatics* 27 2957–2963. 10.1093/bioinformatics/btr507 21903629 PMC3198573

[B51] Mahadevan A. (2016). The impact of submesoscale physics on primary productivity of plankton. *Ann. Rev. Mar. Sci.* 8 161–184. 10.1146/annurev-marine-010814-015912 26394203

[B52] Marshall K. T. Morris R. M. (2013). Isolation of an aerobic sulfur oxidizer from the SUP05/Arctic96BD-19 clade. *ISME J.* 7 452–455. 10.1038/ismej.2012.78 22875135 PMC3554405

[B53] Martínez-Pérez C. Greening C. Bay S. K. Lappan R. J. Zhao Z. De Corte D. (2022). Phylogenetically and functionally diverse microorganisms reside under the Ross Ice Shelf. *Nat. Commun.* 13:117. 10.1038/s41467-021-27769-5 35013291 PMC8748734

[B54] Mattes T. E. Ingalls A. E. Burke S. Morris R. M. (2021). Metabolic flexibility of SUP05 under low DO growth conditions. *Environ. Microbiol.* 23 2823–2833. 10.1111/1462-2920.15226 32893469 PMC7936001

[B55] Mehrshad M. Rodriguez-Valera F. Amoozegar M. A. López-García P. Ghai R. (2018). The enigmatic SAR202 cluster up close: shedding light on a globally distributed dark ocean lineage involved in sulfur cycling. *ISME J.* 12 655–668. 10.1038/s41396-017-0009-5 29208946 PMC5864207

[B56] Mena C. Reglero P. Balbín R. Martín M. Santiago R. Sintes E. (2020). Seasonal niche partitioning of surface temperate open ocean prokaryotic communities. *Front. Microbiol.* 11:1749. 10.3389/fmicb.2020.01749 32849378 PMC7399227

[B57] Mena C. Reglero P. Balbín R. Martín M. Santiago R. Sintes E. (2022). Dynamics of actively dividing prokaryotes in the western Mediterranean Sea. *Sci. Rep.* 12:2064. 10.1038/s41598-022-06120-y 35136122 PMC8825817

[B58] Moreno-Ulloa A. Sicairos Diaz V. Tejeda-Mora J. A. Macias Contreras M. I. Castillo F. D. Guerrero A. (2020). Chemical profiling provides insights into the metabolic machinery of hydrocarbon-degrading deep-sea microbes. *mSystems* 5:e00824-20. 10.1128/mSystems.00824-20 33172970 PMC7657597

[B59] Morris R. M. Frazar C. D. Carlson C. A. (2012). Basin-scale patterns in the abundance of SAR11 subclades, marine *Actinobacteria* (OM1), members of the *Roseobacter clade* and OCS116 in the South Atlantic. *Environ. Microbiol.* 14 1133–1144. 10.1111/j.1462-2920.2011.02694.x 22225975

[B60] Morris R. M. Rappé M. S. Urbach E. Connon S. A. Giovannoni S. J. (2004). Prevalence of the Chloroflexi-related SAR202 bacterioplankton cluster throughout the mesopelagic zone and deep ocean. *Appl. Environ. Microbiol.* 70 2836–2842. 10.1128/aem.70.5.2836-2842.2004 15128540 PMC404461

[B61] Mußmann M. Pjevac P. Krüger K. Dyksma S. (2017). Genomic repertoire of the Woeseiaceae/JTB255, cosmopolitan and abundant core members of microbial communities in marine sediments. *ISME J.* 11 1276–1281. 10.1038/ismej.2016.185 28060363 PMC5437919

[B62] Oschlies A. (2021). A committed fourfold increase in ocean oxygen loss. *Nat. Commun.* 12:2307. 10.1038/s41467-021-22584-4 33863893 PMC8052459

[B63] Padilla C. C. Bristow L. A. Sarode N. Garcia-Robledo E. Gómez Ramírez E. Benson C. R. (2016). NC10 bacteria in marine oxygen minimum zones. *ISME J.* 10 2067–2071. 10.1038/ismej.2015.262 26918666 PMC5029155

[B64] Pajares S. Varona-Cordero F. Hernández-Becerril D. U. (2020). Spatial distribution patterns of bacterioplankton in the oxygen minimum zone of the Tropical Mexican Pacific. *Microb. Ecol.* 80 519–536. 10.1007/s00248-020-01508-7 32415330

[B65] Paulmier A. Ruiz-Pino D. (2009). Oxygen minimum zones (OMZs) in the modern ocean. *Prog. Oceanogr.* 80 113–128. 10.1016/j.pocean.2008.08.001

[B66] Penn J. L. Weber T. Chang B. X. Deutsch C. (2019). Microbial ecosystem dynamics drive fluctuating nitrogen loss in marine anoxic zones. *Proc. Natl. Acad. Sci. U.S.A.* 116 7220–7225. 10.1073/pnas.1818014116 30910952 PMC6462081

[B67] Rajpathak S. N. Roumik B. Mishra P. G. Khedkar A. M. Patil Y. M. Joshi S. R. (2018). An exploration of microbial and associated functional diversity in the OMZ and non-OMZ areas in the Bay of Bengal. *J. Biosci.* 43 635–648. 10.1007/s12038-018-9781-2 30207310

[B68] Reintjes G. Tegetmeyer H. E. Bürgisser M. Orlić S. Tews I. Zubkov M. (2019). On-Site analysis of bacterial communities of the ultraoligotrophic South Pacific Gyre. *Appl. Environ. Microbiol.* 85:e00184-19. 10.1128/AEM.00184-19 31076426 PMC6606877

[B69] Rinke C. Schwientek P. Sczyrba A. Ivanova N. N. Anderson I. J. Cheng J. F. (2013). Insights into the phylogeny and coding potential of microbial dark matter. *Nature* 499 431–437. 10.1038/nature12352 23851394

[B70] Schloss P. D. Westcott S. L. Ryabin T. Hall J. R. Hartmann M. Hollister E. B. (2009). Introducing mothur: open-source, platform-independent, community-supported software for describing and comparing microbial communities. *Appl. Environ. Microbiol.* 75 7537–7541. 10.1128/aem.01541-09 19801464 PMC2786419

[B71] Schmidtko S. Stramma L. Visbeck M. (2017). Decline in global oceanic oxygen content during the past five decades. *Nature* 542 335–339. 10.1038/nature21399 28202958

[B72] Schwalbach M. S. Tripp H. J. Steindler L. Smith D. P. Giovannoni S. J. (2010). The presence of the glycolysis operon in SAR11 genomes is positively correlated with ocean productivity. *Environ. Microbiol.* 12 490–500. 10.1111/j.1462-2920.2009.02092.x 19889000

[B73] Segata N. Izard J. Waldron L. Gevers D. Miropolsky L. Garrett W. S. (2011). Metagenomic biomarker discovery and explanation. *Genome Biol.* 12:R60. 10.1186/gb-2011-12-6-r60 21702898 PMC3218848

[B74] Shah V. Chang B. X. Morris R. M. (2017). Cultivation of a chemoautotroph from the SUP05 clade of marine bacteria that produces nitrite and consumes ammonium. *ISME J.* 11 263–271. 10.1038/ismej.2016.87 27434424 PMC5315479

[B75] Sheik C. S. Jain S. Dick G. J. (2014). Metabolic flexibility of enigmatic SAR324 revealed through metagenomics and metatranscriptomics. *Environ. Microbiol.* 16 304–317. 10.1111/1462-2920.12165 23809230

[B76] Sheremet A. Jones G. M. Jarett J. Bowers R. M. Bedard I. Culham C. (2020). Ecological and genomic analyses of candidate phylum WPS-2 bacteria in an unvegetated soil. *Environ. Microbiol.* 22 3143–3157. 10.1111/1462-2920.15054 32372527

[B77] Sun X. Kop L. F. M. Lau M. C. Y. Frank J. Jayakumar A. Lücker S. (2019). Uncultured Nitrospina-like species are major nitrite oxidizing bacteria in oxygen minimum zones. *ISME J.* 13 2391–2402. 10.1038/s41396-019-0443-7 31118472 PMC6776041

[B78] Suter E. A. Pachiadaki M. Taylor G. T. Astor Y. Edgcomb V. P. (2018). Free-living chemoautotrophic and particle-attached heterotrophic prokaryotes dominate microbial assemblages along a pelagic redox gradient. *Environ. Microbiol.* 20 693–712. 10.1111/1462-2920.13997 29160034

[B79] Thrash J. C. Seitz K. W. Baker B. J. Temperton B. Gillies L. E. Rabalais N. N. (2017). Metabolic roles of uncultivated bacterioplankton lineages in the Northern Gulf of Mexico “Dead Zone”. *mBio* 8:e01017-17. 10.1128/mBio.01017-17 28900024 PMC5596340

[B80] Tsementzi D. Wu J. Deutsch S. Nath S. Rodriguez R. L. Burns A. S. (2016). SAR11 bacteria linked to ocean anoxia and nitrogen loss. *Nature* 536 179–183. 10.1038/nature19068 27487207 PMC4990128

[B81] Ulloa O. Canfield D. E. DeLong E. F. Letelier R. M. Stewart F. J. (2012). Microbial oceanography of anoxic oxygen minimum zones. *Proc. Natl. Acad. Sci. U.S.A.* 109 15996–16003. 10.1073/pnas.1205009109 22967509 PMC3479542

[B82] van Vliet D. M. von Meijenfeldt F. A. B. Dutilh B. E. Villanueva L. Sinninghe Damsté J. S. Stams A. J. M. (2021). The bacterial sulfur cycle in expanding dysoxic and euxinic marine waters. *Environ. Microbiol.* 23 2834–2857. 10.1111/1462-2920.15265 33000514 PMC8359478

[B83] Vaquer-Sunyer R. Duarte C. M. (2008). Thresholds of hypoxia for marine biodiversity. *Proc. Natl. Acad. Sci. U.S.A.* 105 15452–15457. 10.1073/pnas.0803833105 18824689 PMC2556360

[B84] Vijayan J. Kumar V. Ammini P. (2020). Comparison of bacterial community structure in coastal and offshore waters of the Bay of Bengal, India. *Reg. Stud. Mar. Sci.* 39:101414. 10.1016/j.rsma.2020.101414

[B85] Walsh D. Zaikova E. Howes C. G. Song Y. Wright J. Tringe S. (2009). Metagenome of a versatile chemolithoautotroph from expanding oceanic dead zones. *Science* 326 578–582. 10.1126/science.1175309 19900896

[B86] Wang B. Wang Z. Wang S. Qiao X. Gong X. Gong Q. (2020). Recovering partial nitritation in a PN/A system during mainstream wastewater treatment by reviving AOB activity after thoroughly inhibiting AOB and NOB with free nitrous acid. *Environ. Int.* 139:105684. 10.1016/j.envint.2020.105684 32247103

[B87] Ward L. M. Cardona T. Holland-Moritz H. (2019). Evolutionary implications of anoxygenic phototrophy in the bacterial phylum *Candidatus* Eremiobacterota (WPS-2). *Front. Microbiol.* 10:1658. 10.3389/fmicb.2019.01658 31396180 PMC6664022

[B88] Wright J. J. Konwar K. M. Hallam S. J. (2012). Microbial ecology of expanding oxygen minimum zones. *Nat. Rev. Microbiol.* 10 381–394. 10.1038/nrmicro2778 22580367

[B89] Wright J. J. Mewis K. Hanson N. W. Konwar K. M. Maas K. R. Hallam S. J. (2014). Genomic properties of Marine Group A bacteria indicate a role in the marine sulfur cycle. *ISME J.* 8 455–468. 10.1038/ismej.2013.152 24030600 PMC3906813

[B90] Zakem E. J. Mahadevan A. Lauderdale J. M. Follows M. J. (2020). Stable aerobic and anaerobic coexistence in anoxic marine zones. *ISME J.* 14 288–301. 10.1038/s41396-019-0523-8 31624350 PMC6908664

[B91] Zhang R. Liu W. C. Liu Y. Zhang H. L. Zhao Z. H. Zou L. Y. (2021). Impacts of anthropogenic disturbances on microbial community of coastal waters in Shenzhen, South China. *Ecotoxicology* 30 1652–1661. 10.1007/s10646-020-02297-y 33161467

[B92] Zhou Z. Meng H. Gu W. Li J. Deng M. Gu J. D. (2022). High-throughput sequencing reveals the main drivers of niche-differentiation of bacterial community in the surface sediments of the northern South China sea. *Mar. Environ. Res.* 178:105641. 10.1016/j.marenvres.2022.105641 35594805

