# Supplementary material for: Microbial community structures and important taxa across oxygen gradients in the Andaman Sea and eastern Bay of Bengal epipelagic waters
Source: Front Microbiol. 2022 Nov 2;13:1041521. doi: 10.3389/fmicb.2022.1041521 (PMC9667114; doi:10.3389/fmicb.2022.1041521)
Supplement: Supplementary file 2 [file Data_Sheet_2.docx]

**Supplementary Figs**


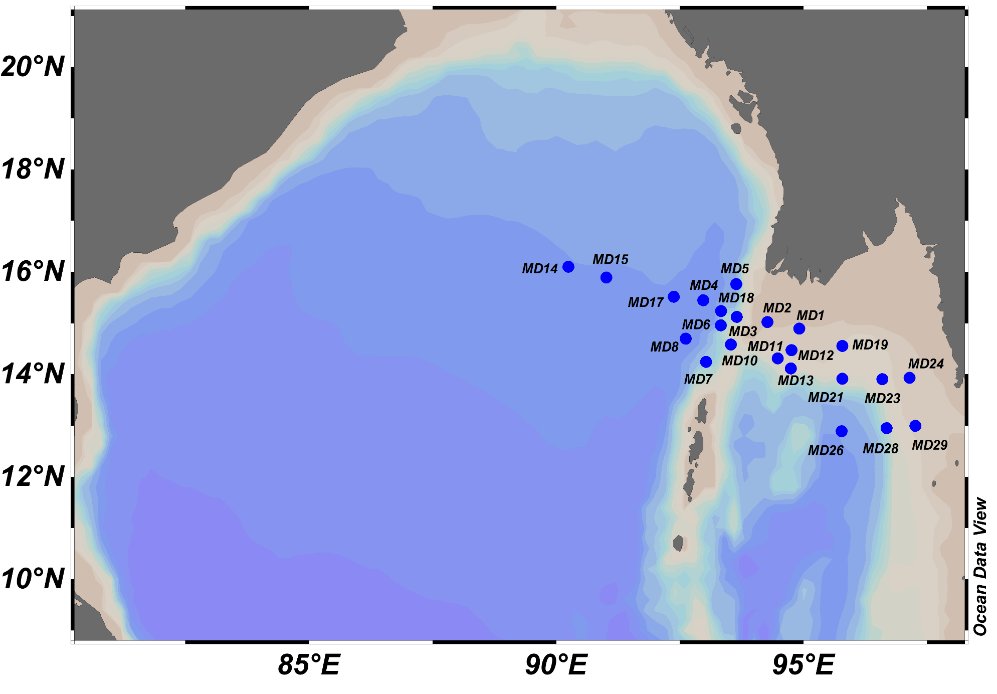


**FIGURE S1|** Map of the study region in the Andaman Sea and eastern Bay of Bengal showing locations of sampling stations.


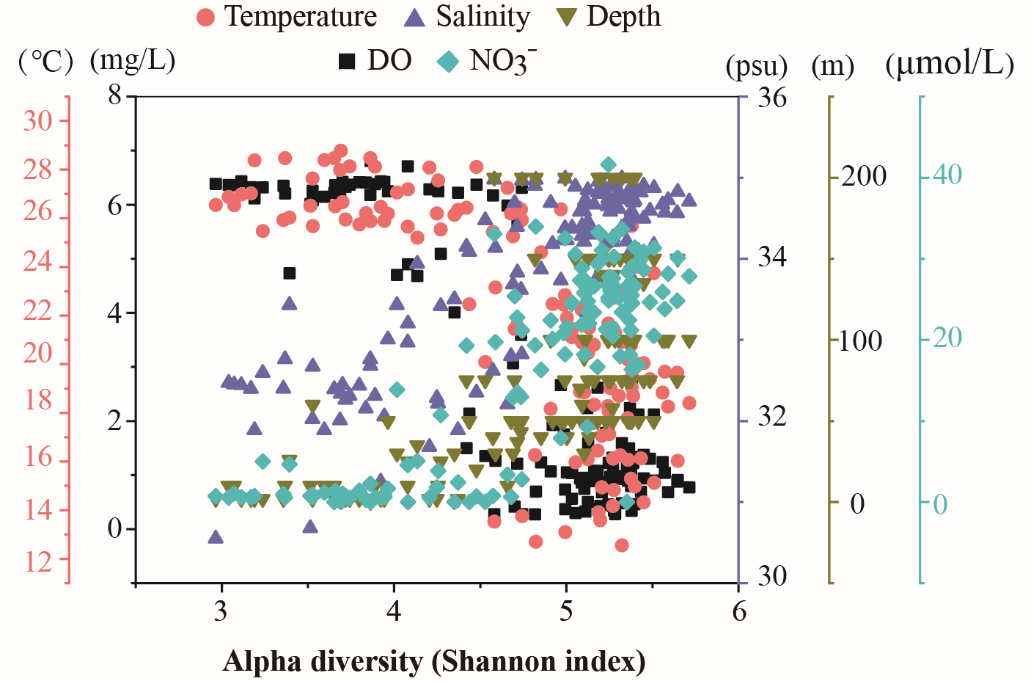


**FIGURE S2|** Spearman correlation analysis of relationships between microbial communities and environmental factors.


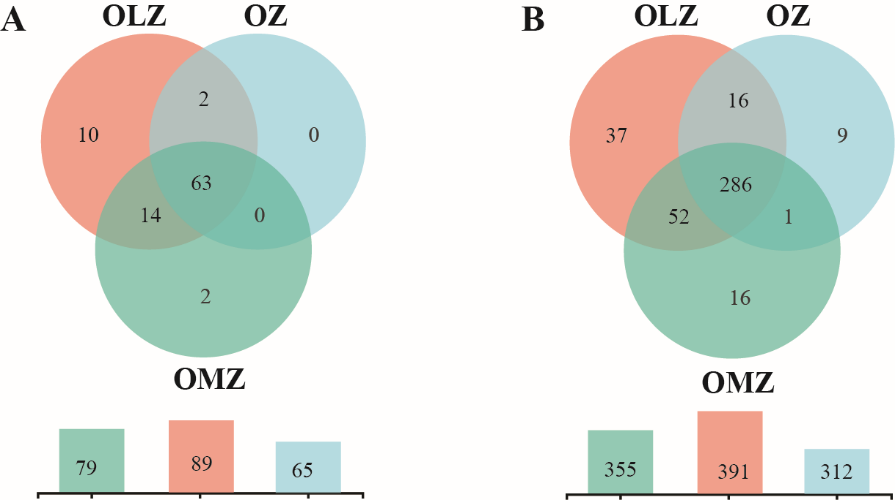


**FIGURE S3|** Venn diagram of microbial community overlap in the OMZ, OLZ, and OZ sites of this study. Diagrams are shown at the (A) class and (B) family level.


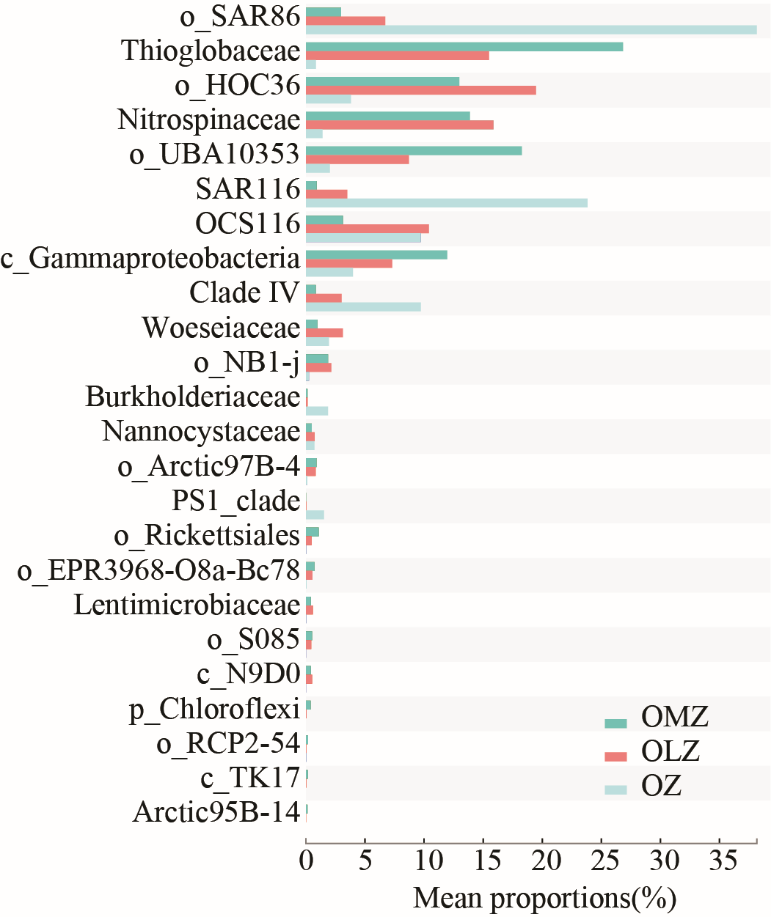


**FIGURE S4|** Mean proportions of 24 most important biomarker taxa selected by random forest model analysis.


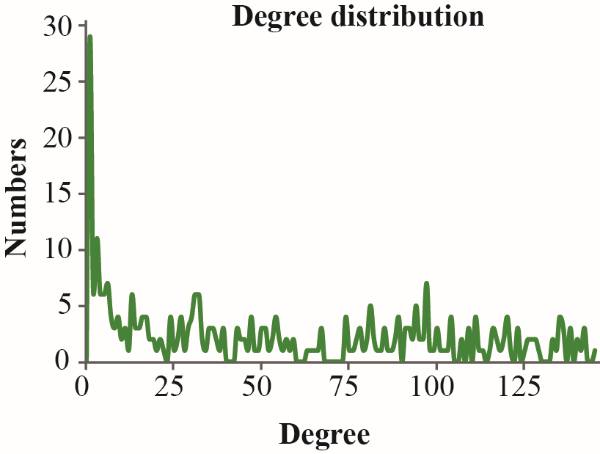


**FIGURE S5|** The distribution of co-occurrence network degrees of microbial taxa.


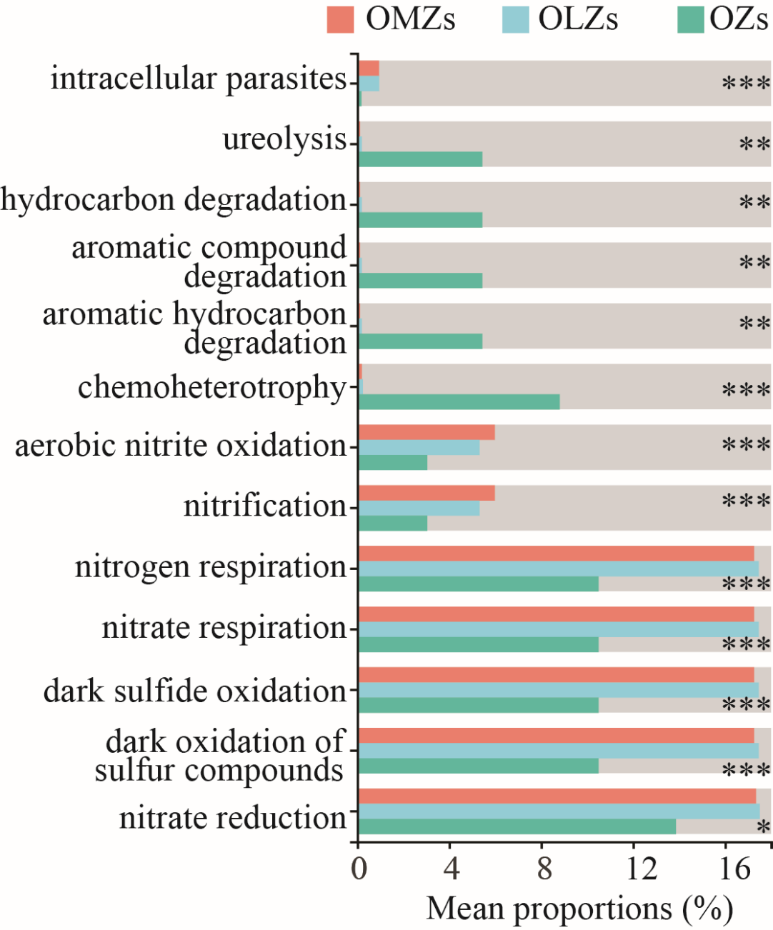


**FIGURE S6|** Functional Annotation of Prokaryotic Taxa (FAPROTAX) functional predictions for the 24 most important biomarker taxa selected by random forest model analysis.


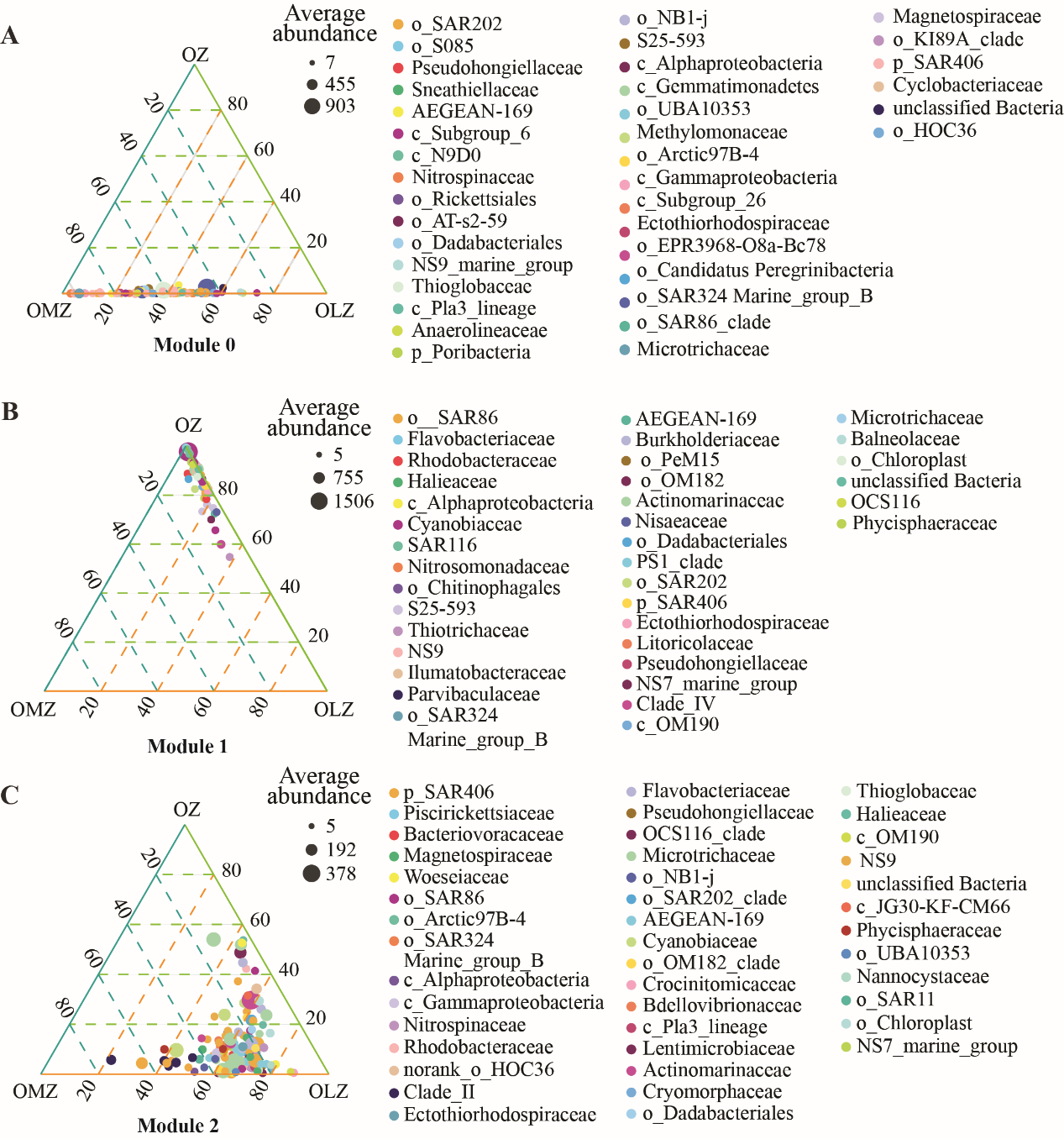


**FIGURE S7|** Ternary plot of OTU distributions from the major modules of the co-occurrence network analysis at the family level. Plots are shown for (A) Module 0, (B) Module 1, and (C) Module 2.

**TABLE S1.** Sampling location and sequencing information.

**TABLE S2.** Sampling stations, depths, and DO ranges.

**TABLE S3.** Permutational Multivariate Analysis of Variance (PERMANOVA) for environmental factors and community composition.

**TABLE S4.** Specific families identified in each group.

**TABLE S5.** Microbial community interaction network nodes and topological properties. The keystone taxa were marked in green.

**TABLE S6.** Spearman’s correlation of the relative abundances of important taxa with environmental factors.
